# Two new species of *Verruconis* from Hainan, China

**DOI:** 10.3897/mycokeys.48.32147

**Published:** 2019-03-05

**Authors:** Min Qiao, Weiguang Tian, Rafael F. Castañeda-Ruiz, Jianping Xu, Zefen Yu

**Affiliations:** 1 Laboratory for Conservation and Utilization of Bio-resources, Key Laboratory for Microbial Resources of the Ministry of Education, Yunnan University, Kunming, Yunnan 650091, China; 2 School of Life Science, Yunnan University, Kunming, China; 3 Instituto de Investigaciones Fundamentales en Agricultura Tropical “Alejandro de Humboldt”, Calle 1 Esq. 2, Santiago, de Las Vegas, Cuba; 4 Department of Biology, McMaster University, Hamilton, Ontario, L8S 4K1, Canada

**Keywords:** Aquatic hyphomycetes, dematiaceous fungi, phylogenetic placement, new taxon

## Abstract

Two new species of the genus *Verruconis*, *V.hainanensis* and *V.pseudotricladiata*, were described using combined morphological and DNA sequence data. The DNA sequences of respective strains including nuclear ribosomal DNA genes (nuSSU, ITS, nuLSU) and fragments of three protein-coding genes (ACT1, BT2, TEF1) were sequenced and compared with those from closely-related species to genera *Ochroconis* and *Verruconis* (Family Sympoventuriaceae, Order Venturiales). Morphologically, both species showed typical ampulliform conidiophores and conidiogenous cells, features not seen in other species of *Verruconis*. The conidia of *V.hainanensis* are fusiform and those of *V.pseudotricladiata* are Y or T shaped, similar to old members of a closely-related genus *Scolecobasidium*. The addition of these two new species provides a new perspective on the heterogeneity of *Scolecobasidium*.

## Introduction

The genus *Verruconis* Samerp. et al. was proposed for the neurotropic opportunist *Ochroconisgallopava* (W.B. Cooke) de Hoog (Samerpitak et al. 2014). The thermophilic characteristic of this genus is remarkable because all three proposed species of *Verruconis* can grow at 35–42 °C. In addition to the difference in growth temperature, *Verruconis* and *Ochroconis* de Hoog & Arx also differed in conidia colour (Samerpitak et al. 2014). However, a recent molecular phylogenetic analysis placed the mesophilic *V.panacis* T. Zhang & Y. Zhang into *Verruconis*, a result suggesting that both genera are more heterogeneous in their morphological and growth requirements than previously thought (Zhang et al. 2018).

Besides *V.panacis*, other three *Verruconis* species were transferred from other genera. The type species, *V.gallopava* (W.B. Cooke) Samerp. & de Hoogs [≡ *Dactylariagallopava* (W.B. Cooke) G.C. Bhatt & W.B. Kendr., ≡ *Ochroconisgallopava* (W.B. Cooke) de Hoog] was transferred from *Diplorhinotrichum* Höhn.; *V.verruculosa* (R.Y. Roy et al.) Samerp. & de Hoog (≡ *Scolecobasidiumverruculosum* R.Y. Roy et al.) was transferred from *Scolecobasidium* and *V.calidifluminalis* (Yarita et al.) Samerp. & de Hoog (≡ *Ochroconiscalidifluminalis* Yarita et al.) was transferred from *Ochroconis*. These reclassifications suggested that genera *Ochroconis*, *Verruconis* and *Scolecobasidium* E.V. Abbott are closely related and that both morphological and molecular data are needed in order to derive robust classifications. *Ochroconis*, typified by *O.constricta* (E.V. Abbott) de Hoog & Arx, transferred from *Scolecobasidium*, was set up to comprise species with unbranched, subspherical to cylindrical or clavate conidia. Based on these criteria, many *Scolecobasidium* species were transferred to *Ochroconis*, while species in the genus *Scolecobasidium* were restricted to those with T- or Y-shaped or bi-lobed, two- to many-celled conidia and ampulliform conidiogenous cells, possessing one to three conidium-bearing denticles at the apex of the conidiogenous cells (de Hoog and von Arx 1973). However, there is a significant disagreement amongst mycologists about whether the genus *Ochroconis* should be established and some researchers still placed species with unbranched conidia under *Scolecobasidium* (Ellis 1976; Matsushima 1980, 1985, 1987, 1993, 1996; Punithalingam and Spooner 2011; Lu et al. 2013; Ren et al. 2013; Xu et al. 2014).

Samerpitak et al. (2014) revised the genera *Ochroconis* and *Scolecobasidium* using DNA sequences of the nuclear ribosomal RNA gene clusters and three protein-coding genes (actin: ACT1, β-tubulin: BT2, translation elongation factor 1-α: TEF1). They found that the type species of *Scolecobasidium*, *S.terreum* E.V. Abbott, ex-type strain CBS 203.27, originally described as having the T-shaped conidia, had lost the ability to produce conidia. Interestingly, this strain was phylogenetically distant from other strains with Y-shaped conidia as described for *S.terreum* in all analyses. Consequently, type strain *S.terreum* CBS 203.27 is now regarded as a non-representative strain of the species and, indeed, the validity of this species has been questioned and *Scolecobasidium* is considered to be of doubtful identity.

However, Gams thought that an ex-type culture was not so important to decide if a genus is retained, because there are other cultures of *S.terreum* available all over the world, which clearly define the identity of this characteristic fungus. He even thought that CBS 510.71, the ex-type of *Humicolaminima* Fassat., a species with characteristic Y-shaped conidia, may replace *S.terreum* (Gams 2015). However, in Samerpitak’s analysis, many *Scolecobasidium* species were scattered outside the Family Sympoventuriaceae. Consequently, the genus *Scolecobasidium* has been questioned (Samerpitak et al. 2014). Since then, several new *Ochroconis* species have been described under *Ochroconis* (Giraldo et al. 2014; Samerpitak et al. 2015a; 2015b; 2017; Crous et al. 2016; 2017), while the number of *Scolecobasidium* species has not increased since 2014 (Index Fungorum 2018). Species with forked conidia, similar to *S.terreum*, were also added to *Ochroconis* based on phylogenetic relationships amongst members of Sympoventuriaceae (Giraldo et al. 2014). The strict morphological characters to demarcate *Scolecobasidium* were abandoned in favour of the molecular phylogenetic approach. Subsequent analyses based on combined molecular sequence information, ecological and physiological traits and morphological differences resulted in the establishment of the genus *Verruconis*.

Hainan Province, China is a centre of biodiversity for aquatic hyphomycetes. Since 2015, we have reported several new aquatic hyphomycetes from this area (Guo et al. 2015; Qiao et al. 2017, 2018). During further studies of aquatic hyphomycetes on submerged decaying leaves collected from a stream in Hainan Province, we encountered two fungi which resembled species of *Scolecobasidium*. Based on phylogenetic analyses, we identified that the fungi belonged to *Verruconis*. In this paper, we describe the two fungi as new species and determined their phylogenetic placement based on the combined sequences of SSU, ITS, LSU, BT2, TEF1 and ACT1.

## Materials and methods

### Collection of samples, isolation and characterisation

Submerged dicotyledonous leaves were collected from a stream in Hainan. Samples were collected in zip-lock plastic bags and labelled and then transported to the laboratory. The rotten leaves were cut into several 2–4 × 2–4 cm sized fragments in the laboratory and then spread on to the surface of CMA (20 g cornmeal, 18 g agar, 40 mg streptomycin, 30 mg ampicillin, 1000 ml distilled water) medium for 10 days; a single conidium was isolated and cultivated on CMA in Petri plates using sterilised needles while viewing with a BX51 microscope. Morphological observations were then made from CMA after incubation at 28 °C for one week. Measurement data were based on 30 random conidia and 10 conidiophores. Pure cultures were deposited in the Herbarium of the Laboratory for Conservation and Utilization of Bio-Resources, Yunnan University, Kunming, Yunnan, P.R. China (YMF, formerly Key Laboratory of Industrial Microbiology and Fermentation Technology of Yunnan) and at the China General Microbiological Culture Collection Center (CGMCC).

### DNA extraction, PCR and sequencing

Total DNA was extracted from fresh mycelia as described by Turner et al. (1997). Six markers, nuSSU, D1/D2 region of nuLSU, ITS and part of ACT1, BT2 and TEF1 were amplified by PCR using primers as reported earlier (Feng et al. 2013). PCR amplifications were performed using the methods described previously (Wang et al. 2014). The PCR products were then sent to the Beijing Tsingke Biotechnology Co. of China Ltd and sequenced on both strands with the same primers that were used for amplification.

### Sequence alignment and phylogenetic analysis

Preliminary BLAST searches with nuSSU and nuLSU gene sequences of the new isolates indicated that they had a close phylogenetic relationship with sequences from the genus *Verruconis*, *Ochroconis* and *Scolecobasidium*. Based on this, we downloaded sequences at the six marker loci from strains belonging to genera *Ochroconis* and *Verruconis*, including 42 strains representing 21 species of *Ochroconis* and four species of *Verruconis*. The sequences of these representative strains were combined with those from our own cultures (see Table 1 for all GenBank accession numbers). *Scolecobasidiumexcentricum* R.F. Castañeda, W. Gams & Saikawa was specified as an outgroup.

**Table 1.** Species, strains and their corresponding GenBank accession numbers of sequences used for phylogenetic analyses.

Taxon	strain	GenBank accession number					
		ACT	BT2	ITS	LSU	SSU	TEF1
*Ochroconisanellii* (Graniti) de Hoog & Arx	CBS 284.64*	KF155912	KF156184	FR832477	KF156138	KF156070	KF155995
*O.anomala* A. Nováková & Mart.-Sánch.	CBS 131816*	KF155935	KF156194	HE575201	KF156137	KF156065	KF155986
*O.constricta* (E.V. Abbott) de Hoog & Arx	CBS 211.53*	KF155941	KF156187	HQ667519	KF156148	KF156073	KF156005
	CBS 202.27	KF155942	KF156161	AB161063	KF156147	KF156072	KF156003
	CBS 269.61	KF155939	KF156163	KF156024	KF156149	KF156074	KF156004
*O.cordanae* Samerp., Crous & de Hoog	CBS 475.80*	HQ916976	KF156197	KF156022	KF156122	KF156058	KF155981
	CBS 172.74	KF155906	KF156198	KF156023	KF156121	KF156057	JF440566
	CBS 780.83	KF155905	KF156199	HQ667539	KF156120	KF156059	KF155979
*O.crassihumicola* (Matsush.) de Hoog & Arx	CBS 120700	KJ867427	KJ867433	KJ867429	KJ867430	KJ867431	KJ867428
*O.gamsii* de Hoog	CBS 239.78*	KF155936	KF156190	KF156019	KF156150	KF156088	KF155982
	CBS 101179	KF155937	KF156192	KF156020	KF156151	KF156091	–
*O.globalis* Samerp., A.P.M. Duarte, Attili-Angelis & de Hoog	CBS 119644*	KF956086	KF961065	KF961086	KF961097	KF961108	KF961075
	CBS 131956	KF956094	KF961067	KF961088	KF961100	KF961117	KF961081
	CBS 135766	KF956087	KF961072	KF961094	KF961106	KF961116	KF961082
*O.humicola* (G.L. Barron & L.V. Busch) de Hoog & Arx	CBS 116655*	KF155904	KF156195	HQ667521	KF156124	KF156068	KF155984
*O.icarus* Samerp., A. Giraldo, Guarro & de Hoog	CBS 116645	LM644599	LM644604	HQ667525	LM644565	KF156083	–
*O.lascauxensis* A. Nováková & Mart.-Sánch.	CBS 131815*	KF155911	KF156183	FR832474	KF156136	KF156069	KF155994
*O.longiphorum* (Matsush.) Samerp. & de Hoog	CBS 435.76*	KF155908	KF156182	KF156038	KF156135	KF156060	KF155978
*O.macrozamiae* Crous & R.G. Shivas	CBS 102491	KF155938	KF156191	KF156021	KF156152	KF156092	KF155983
*O.minima* (Fassat.) Samerp. & de Hoog	CBS 423.64	KF155943	KF156173	HQ667523	KF156131	KF156085	KF156008
	CBS 536.69	KF155944	KF156174	HQ667524	KF156132	KF156084	KF156009
*O.mirabilis* Samerp. & de Hoog	CBS 413.51	KF155957	KF156164	HQ667536	KF156140	KF156076	KF156001
	dH 14815	KF155954	KF156170	KF156036	KF156145	KF156079	KF155998
*O.musae* (G.Y. Sun & Lu Hao) Samerp. & de Hoog	CBS 729.95*	KF155948	KF156171	KF156029	KF156144	KF156082	KF155999
*O.ramosa* A. Giraldo, Gené, Deanna A. Sutton & Guarro	UTHSC 03-3677	LM644601	LM644606	LM644522	LM644566	LM644549	–
	UTHSC 04-2729	LM644602	LM644607	LM644523	LM644567	LM644550	–
	UTHSC 12-1082	LM644603	LM644608	LM644524	–	LM644551	–
*O.sexualis* Samerp., Van der Linde & de Hoog	dH 22953	KF155903	KF156188	KF156017	KF156119	KF156090	KF155977
	PPRI 12991*	KF155902	KF156189	KF156018	KF156118	KF156089	KF155976
*O.tshawytschae* (Doty & D.W. Slater) Kiril. & Al-Achmed	CBS 130.65	KF155916	KF156178	HQ667566	KF156127	KF156061	KF155989
	CBS 228.66	KF155915	KF156179	KF156016	KF156128	KF156064	KF155992
	CBS 100438*	KF155918	KF156180	HQ667562	KF156126	KF156062	KF155990
*O.verrucosa* (Zachariah, Sankaran & Leelav.) Samerp. & de Hoog	CBS 225.77	KF155909	KF156186	–	KF156130	KF156066	KF155985
	CBS 383.81*	KF155910	KF156185	KF156015	KF156129	KF156067	–
*Scolecobasidiumexcentricum* R.F. Castañeda, W. Gams & Saikawa	CBS 469.95*	KF155934	KF156196	HQ667543	KF156105	KF156096	KF155975
*Verruconiscalidifluminalis* (Yarita, A. Sano, de Hoog & Nishim.) Samerp. & de Hoog	CBS 125818*	KF155901	KF156202	AB385698	KF156108	KF156046	KF155959
*V.gallopava* (W.B. Cooke) Samerp. & de Hoog	CBS 437.64*	HQ916989	KF156203	HQ667553	KF156112	KF156053	KF155968
	CBS 118.91	–	–	–	KF156110	KF156047	–
	CBS 863.95	–	–	–	KF156114	KF156052	–
*Verruconisverruculosa* (R.Y. Roy, R.S. Dwivedi & R.R. Mishra) Samerp. & de Hoog	CBS 119775*	KF155919	KF156193	KF156014	KF156106	KF156055	KF155974
*Verruconishainanensis* Z.F. Yu & M. Qiao	YMF1.04165*	**MK248271**	–	**MK244397**	**MK248269**	MF536879	MF536881
*Verruconispanacis* T. Zhang & Y. Zhang	SYPF8337*	–	MF536883	MF536882	MF536880	**MK248267**	**MK248272**
*Verruconispseudotricladiata* Z.F. Yu & M. Qiao	YMF1.04915*	–	**MK253013**	**MK244396**	**MK248270**	**MK248268**	**MK248273**

Note: Numbers in bold are those generated in this study. Marked with * are type strains.

Six alignment files were generated, one for each gene and converted to NEXUS files with ClustalX 1.83 (Thompson et al. 1997) to identify the phylogenetic positions of two species. The six alignments were then combined with BioEdit 7.1.9.0 (Hall 1999). All characters were weighted equally and gaps were treated as missing characters. Maximum likelihood (ML) analysis was computed by RAxML (Stamatakis 2006) with the PHY files generated with ClustalX 1.83 (Thompson et al. 1997), using the GTR-GAMMA model. Maximum likelihood bootstrap proportions (MLBP) were computed with 1000 replicates. Bayesian inference (BI) analysis was conducted with MrBayes v3.2.2 (Ronquist et al. 2012). The Akaike information criterion (AIC) implemented in jModelTest 2.0 (Posada 2008) was used to select the best fit models after likelihood score calculations were done. The base tree for likelihood calculations was ML-optimised. HKY+I+G was estimated as the best-fit model under the output strategy of AIC, Metropolis-coupled Markov chain Monte Carlo (MCMCMC) searches were run for 2000000 generations, sampling every 1000^th^ generation. Two independent analyses with four chains each (one cold and three heated) were run until the average standard deviation of the split frequencies dropped below 0.01. The initial 25% of the generations of MCMC sampling were discarded as burn-in. The refinement of the phylogenetic tree was used for estimating Bayesian inference posterior probability (BIPP) values. The Tree was viewed in FigTree v1.4. The values of Maximum likelihood bootstrap proportions (MLBP) greater than 70% and Bayesian inference posterior probabilities (BIPP) greater than 0.95 at the nodes are shown along branches.

## Results

### Phylogenetic analysis

**Figure 1.**
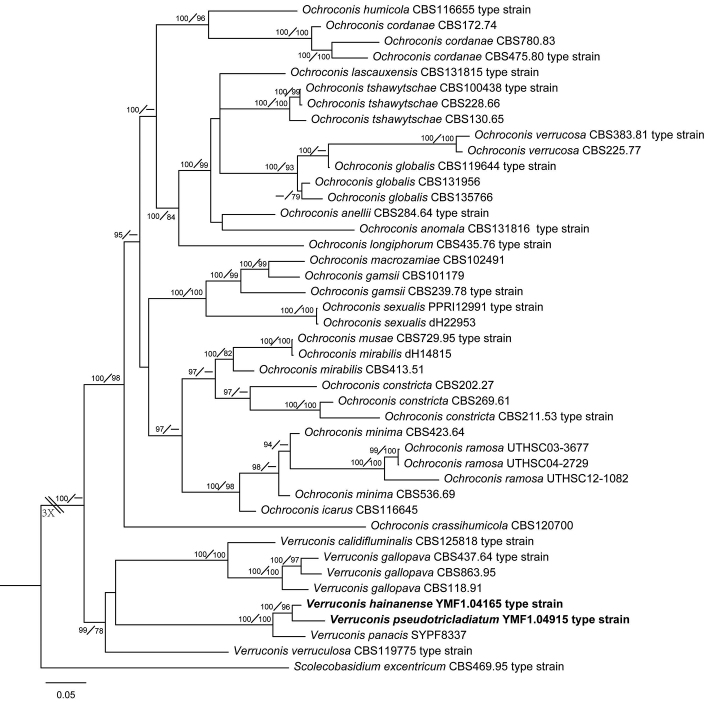
Phylogenetic tree based on Bayesian analysis of the combined sequences of SSU, ITS, LSU BT2, TEF1 and ACT1. *Scolecobasidiumexcentricum* is used as the outgroup. Bayesian posterior probabilities, greater than 0.95, are given above the nodes. Maximum likelihood bootstrap values, greater than 75%, are given below the nodes. The scale bar shows the expected changes per site.

The phylogenetic relationships amongst the known representative taxa are completely congruent with the previous studies (Samerpitak et al. 2014; Giraldo et al. 2014). *Ochroconis* and *Verruconis* formed two distinct clades. Within the *Ochroconis* clade, three species, *O.minima* (Fassat.) Samerp. & de Hoog, *O.ramose* A. Giraldo et al., *O.icarus* Samerp.et al. with T-shaped conidia fell into a highly-supported sub-clade. Both *V.hainanensis* and *V.pseudotricladiata* were nested in a well -supported subclade, with *V.panacis* as the closest sister species. The sub-clade comprising the two new species and *V.panacis* is closer to the clade composed of *V.calidifluminalis* and *V.gallopava* than to *V.verruculosa* (Figure 1).

### Taxonomy

#### Verruconis hainanensis

Taxon classificationFungiVenturialesSympoventuriaceae

Z.F. Yu & M. Qiao sp. nov.

MB828550

Figure 2

##### Etymology.

Latin, *hainanensis*, refers to the collection locality.

##### Description.

Colonies on CMA medium compact, restricted, brown to fuliginous, 13 mm at 20 °C after 20 days, 16 mm at 25 °C, 11 mm at 30 °C, no growth at 35 °C. Aerial hyphae subhyaline to brown, smooth- or somewhat rough-walled. *Conidiophores* semi-macronematous, mononematous, sometimes slightly moniliform, unbranched or branched at the apex with 2–4 divergent conidiogenous cells, brown basal cell, pale brown branches, smooth, up to 25 μm long. *Conidiogenous* cells mostly monoblastic, discrete, scattered, brown to fuliginous or pale brown, lageniform to ampulliform, pale brown, 3.4–6.0 × 2.2–3.6 μm, with a fimbriate denticle-like at the conidiogenous locus after rhexolytic conidial secession. *Conidia* solitary, acrogenous, fusiform, rostrate at the apical cell, 3-septate, dark at the septa, coarsely verrucose, more or less equilateral, slightly constricted at the median septum, bicoloured, with brown middle cells and subhyaline end cells, 23–30.2 × 3.6–5.7 μm, with an inconspicuous basal frill.

**Figure 2.**
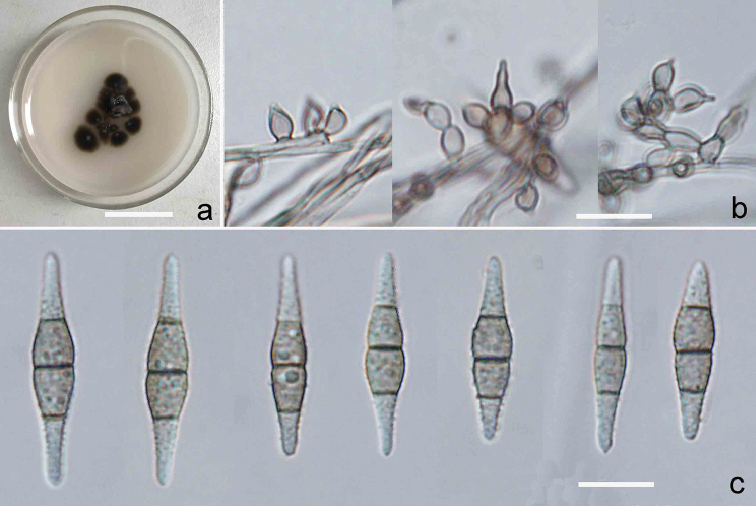
Culture and anamorph of *Verruconishainanensis* (YMF 1.04165). **a** Culture on CMA at 25 °C after 20 days **b** conidiophores and monoblastic conidiogenous cells **c** Conidia; Scale bars: 2 cm (**a**); 10 μm (**b, c**).

##### Type.

**CHINA.** From leaves of an unidentified dicotyledonous plant submerged in a stream, Qixianling, Hainan Province, 18°68'N, 109°69'E, 902 m alt., 16 June 2016, Z.F. Yu (dried slide YMFT 1.04165, holotype; live culture YMF 1.04165 –ex-type culture; CGMCC–3.18974–isotype).

##### Notes.

*Verruconishainanensis* shares the fusiform conidial shape with some described *Scolecobasidium* species, such as: *S.cateniphorum* Matsush., *S.caffrum* Matsush., *S.houhense* D.W. Li & Jing Y. Chen and *S.tropicum* Matsush., but all these taxa are readily distinguishable from the new Chinese species. Specifically, *S.cateniphorum* is distinguished by its 1-septate, smooth or inconspicuous echinulate, 10–24 × 2–3.5 μm conidia (Matsushima 1975). *S.caffrum* and *S.tropicum* both have 2-septate conidia, but *S.caffrum* has conidia mostly smooth or inconspicuously rough, 20–35 × 4–7.5 μm, with pale brown central and subhyaline end cells (Matsushima 1996) and *S.tropicum* has conidia with smooth or inconspicuous verruculose, smaller, 14–20 × 4.5–6 μm, with pale brown central and subhyaline end cells (Matsushima 1983). *S.houhense* with 3-septate conidia is superficially similar to *V.hainanensis*, but *S.houhense* is characterised by minutely verruculose conidia, 26–31 × 4.5–5.5 μm, brown, with central cells darker than end cells and slightly protuberate and with a dark basal scar and its conidiogenous cells and conidiophores are different from those of *V.hainanensis* (Li et al. 2010). The distinct dark scar, described from *S.houhense*, has been reported by Matsushima (1975) in *Nakataeafusispora* (Matsush) Matsush., but it is absent in *V.hainanensis*.

#### Verruconis pseudotricladiata

Taxon classificationFungiVenturialesSympoventuriaceae

Z.F. Yu & M. Qiao sp. nov.

MB828551

Figure 3

##### Etymology.

Latin, *pseudotricladiata* refers to similar conidia shape to *Scolecobasidiumtricladiatum*.

##### Description.

Colonies on CMA medium compact, restricted, brown to fuliginous, surface velvety or floccose, 12 mm at 20 °C after 20 days, 14 mm at 25 °C, 10 mm at 30 °C, no growth at 35 °C. Mycelium subhyaline to pale brown and smooth- or somewhat rough-walled. *Conidiophores* semi-macronematous, mononematous, straight or flexuous, 1–4 septa, sometimes moniliform (composed of 2–5 globose serial cells), pale brown, smooth, 6.5–27.2 × 2.1–3.5 μm, sometimes reduced to conidiogenous cells that arise from assimilative hyphae. *Conidiogenous* cells monoblastic, rarely polyblastic after sympodial elongation, globose, ampulliform, lageniform to clavate, 3.0–5.3 × 2.3–3.8 μm, integrated or discrete, mostly determinate, with an inconspicuous or distinct fimbriate denticle-like at the conidiogenous locus after rhexolytic conidial secession. *Conidia* mostly acrogenous, subhyaline to pale brown, smooth to verruculose, staurosporic, unbranched or branched: i) unbranched conidia (main axis) cylindrical-clavate, 2–4 septate, slightly constricted at the septa, mostly smooth, rarely verruculose, 16–20 × 3.3–4.7 μm, with an inconspicuous basal frill and often with a globose or ellipsoidal, 0–1 septate, 5.6–12.3 × 2.8–4.5 μm primary branch at the apex; ii) branched conidia staurosporic, Y-, or T-shaped, composed of the main axis and two branches (primary and secondary); iia) main axis cylindrical-clavate to clavate, 1–3-septate, mostly 2-septate, smooth or rarely verruculose, very pale brown, 15.6–20.6 × 3.8–5.7 μm; iib) primary branches obclavate, 1–2 septate, verruculose toward the apex, smooth at the basal cell, 17.9–18.2 × 2.9–4.7 μm, at an angle of 45° arising from the apex of main axis; iic) secondary branches ovoid to obclavate, smooth or verruculose towards the apex, 0–2-septate, (–5.6)12.3–17.9 × 2.8–4.5 μm, arising eccentrically from the basal cell of the primary branches.

**Figure 3.**
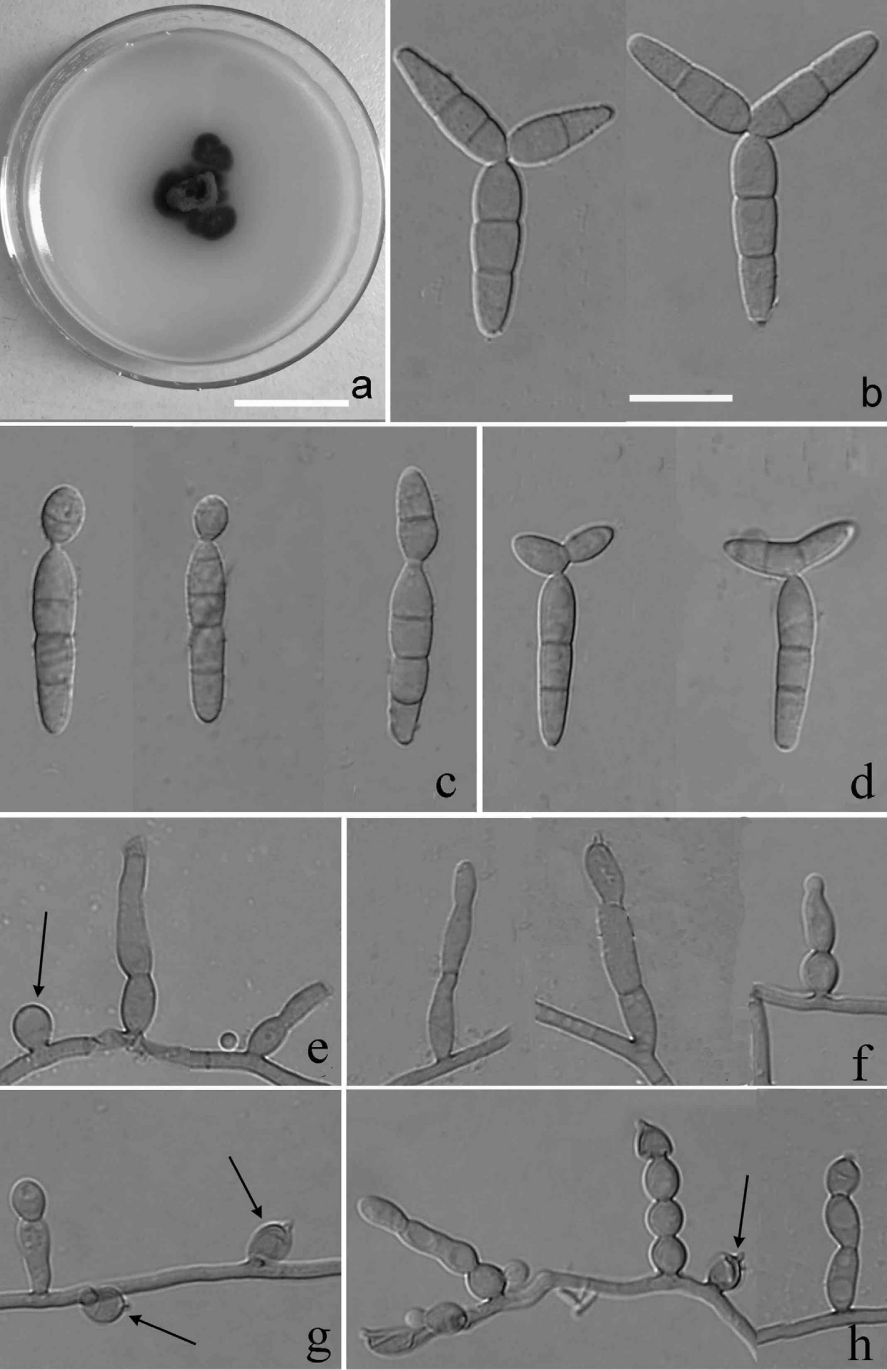
Cultures and anamorph of *Verruconispseudotricladiata* (YMF 1.04915). **a** Cultures on CMA at 25 °C after 20 days **b** branched Y-shaped conidia **c** unbranched conidia **d** T-shaped conidia **h** Conidiophores and conidiogenous cells. Conidiogenous cells on hyphae (black arrow). Scale bars: 2 cm (**a**); 10 μm (**b–h**).

##### Type.

**CHINA.** From leaves of an unidentified broad-leaf species submerged in a stream, Diaoluo Mountain, Hainan Province, 18°41'N, 109°41'E, 254 m alt., 16 June 2016, Z.F. Yu (dried slide YMFT 1.04915, holotype; live culture YMF 1.04915 ex-type; CGMCC–3.18939–isotype).

##### Notes.

*Verruconispseudotricladiata* is similar to *S.tricladiatum* Matsush. on the general conidial morphology, but in *S.tricladiatum*, the conidiophores are mostly moniliform, irregularly branched forming profuse fascicules and, on pure culture, lack staurosporic conidia or rarely formed on the conidiogenous cells, the conidia are mostly unbranched, ellipsoidal to fusiform, (1–) 3–4 (–5)-septate, (9.5–)14–22 (–28) × 4–5 (–6) μm, pale olivaceous or pale brown, verruculose conidia (Matsushima 1971).

## Discussion

The Index Fungorum currently lists 66 names in *Scolecobasidium*. However, 22 of these 66 names have been transferred into genera *Dactylaria* Sacc., *Paradendryphiella* Woudenb. & Crous, *Ochroconis*, *Trichoconis* Clem., *Neta* Shearer & J.L. Crane and *Verruconis* (Index Fungorum 2018). Of the remaining 42 species, the majority lacks authentic culture materials and DNA sequence data, making the revision of *Scolecobasidium* very difficult. However, since 2014, the number of *Scolecobasidium* species has not increased, while many new species have been reported under *Ochroconis*, including species with forked conidia (Giraldo et al. 2014). Although *Scolecobasidium* is still listed as an accepted genus of Ascomycota (Wijayawardene et al. 2017), this genus will likely be phased out. Thus, we have placed our strains into *Verruconis* based on phylogenetic analysis.

Morphologically, the two new species resemble some members of the genus *Scolecobasidium*. Conidiophores composed of 2–5 globose serial cells are very typical in old members of *Scolecobasidium*, such as *S.alabamense* Matsush., *S.amazonense* Matsush., *S.cateniphorum* Matsush. and *S.lanceolatum* Matsush. However, amongst these species, only the LSU sequence of *S.cateniphorum* was available. Further, Y- branched conidia of *V.pseudotricladiata* was previously only described in *S.tricladiatum*, while T-shaped branched conidia appeared in four species, including the type species *S.terreum*, *O.minima* (Fassat.) Samerp. & de Hoog, *O.ramosa* Samerp. et al. and *O.icarus* Samerp. et al. In the molecular phylogenetic tree, inferred from the combined sequences of six marker loci, except for the type species, three species with T-shaped branched conidia form a single clade with high support within *Ochroconis*. In the combined analysis of SSU and LSU, *S.tricladiatum* strain P051 is closely related to *V.pseudotricladiata* and *S.terreum* 043 fell into *Ochroconis*, nested with other species with T-shaped branched conidia (data not shown). The phylogenetic analysis is partly consistent with the morphological comparison. The article, comprising sequences of *S.tricladiatum* strain P051 and *S.terreum* 043, has not been published and we do not know if two species have been identified correctly. Anyhow, molecular data for our strains will help improve the taxonomy and revision of *Scolecobasidium*.

When the genus *Verruconis* was established, the thermophilic character was one of the main characteristics distinguishing this genus from *Ochroconis*. The first three species included in this genus all have a high optimal growing temperature of 35–42 °C and maximum growing temperature of 47–50 °C (Samerpitak et al. 2014). However, both our species and their close relative *V.panacis* are mesophilic, which blurred a major distinguishing feature between *Verruconis* and *Ochroconis*. Morphologically, *Verruconis* is characterised by poorly differentiated, flexible, mostly cylindrical to acicular, with 0(−1) thin septa conidiophores, sometimes without conidiophores (Samerpitak et al. 2014). However, conidiophores of two new species are distinct, only occasionally reducing to conidiogenous cells. Ampulliform conidiogenous cells also appeared in *O.minima* and *O.icarus*, but no species in *Verruconis* and *Ochroconis* have similar conidiophores to those of the two new species, which were composed of 2–5 globose serial cells. Based on the phylogenetic relationships amongst the species, the distributions of morphological features indicate that conidiophores and conidiogenous cells are important features for defining these two related genera. Our results suggest that the analyses of more sequences and more cultures in this group of fungi are needed to provide a robust revision of the three genera *Verruconis*, *Ochroconis* and *Scolecobasidium*.

## Supplementary Material

XML Treatment for Verruconis hainanensis

XML Treatment for Verruconis pseudotricladiata
